# Funding gap for immunization across 94 low- and middle-income countries

**DOI:** 10.1016/j.vaccine.2016.09.036

**Published:** 2016-12-07

**Authors:** Sachiko Ozawa, Simrun Grewal, Allison Portnoy, Anushua Sinha, Richard Arilotta, Meghan L. Stack, Logan Brenzel

**Affiliations:** a Division of Practice Advancement and Clinical Education, UNC Eshelman School of Pharmacy, University of North Carolina - Chapel Hill, Chapel Hill, NC, USA; b Department of Pharmacy, University of Washington, Seattle, WA, USA; c Department of Global Health and Population, Harvard T.H. Chan School of Public Health, Boston, MA, USA; d Department of Preventive Medicine and Community Health, New Jersey Medical School, Rutgers University, Newark, NJ, USA; e Independent Consultant, Denver, CO, USA; f Bill and Melinda Gates Foundation, Washington, DC, USA

**Keywords:** Funding gap, Financing, Vaccine, Low- and middle-income country, Immunization

## Abstract

Novel vaccine development and production has given rise to a growing number of vaccines that can prevent disease and save lives. In order to realize these health benefits, it is essential to ensure adequate immunization financing to enable equitable access to vaccines for people in all communities. This analysis estimates the full immunization program costs, projected available financing, and resulting funding gap for 94 low- and middle-income countries over five years (2016–2020). Vaccine program financing by country governments, Gavi, and other development partners was forecasted for vaccine, supply chain, and service delivery, based on an analysis of comprehensive multi-year plans together with a series of scenario and sensitivity analyses.

Findings indicate that delivery of full vaccination programs across 94 countries would result in a total funding gap of $7.6 billion (95% uncertainty range: $4.6–$11.8 billion) over 2016–2020, with the bulk (98%) of the resources required for routine immunization programs. More than half (65%) of the resources to meet this funding gap are required for service delivery at $5.0 billion ($2.7–$8.4 billion) with an additional $1.1 billion ($0.9–$2.7 billion) needed for vaccines and $1.5 billion ($1.1–$2.0 billion) for supply chain. When viewed as a percentage of total projected costs, the funding gap represents 66% of projected supply chain costs, 30% of service delivery costs, and 9% of vaccine costs. On average, this funding gap corresponds to 0.2% of general government expenditures and 2.3% of government health expenditures.

These results suggest greater need for country and donor resource mobilization and funding allocation for immunizations. Both service delivery and supply chain are important areas for further resource mobilization. Further research on the impact of advances in service delivery technology and reductions in vaccine prices beyond this decade would be important for efficient investment decisions for immunization.

## Highlights

• Estimated financing and funding gap for vaccine, supply chain and service delivery.
• Identified immunization funding gap: $7.6 billion over 2016–20 across 94 countries.
• On average, the funding gap represents 2.3% of government health expenditures.
• Largest funds needed for service delivery and supply chain to meet projected costs.

## 1 Introduction

The cost of national immunization programs (NIPs) is increasing with the introduction of new vaccines and efforts to improve coverage of existing vaccine schedules. A greater need to vaccinate children in more remote and hard-to-reach areas also contributes to the rising costs of immunization. The Global Vaccine Action Plan (GVAP) 2011–2020 – endorsed by the 194 member states of the World Health Assembly in May 2012 – was created to facilitate commitments to support immunization by presenting a roadmap for strengthening NIPs through increased vaccination coverage and introduction of new and underutilized vaccines. It outlines a mission to improve health by extending, to 2020 and beyond, the full benefits of immunization to all people. In order to achieve GVAP goals, understanding the resources required for vaccination programs and the projected gaps in financing is important to ensure adequate and sustainable health investments in the world’s poorest countries.

While governments share in the financing of vaccination programs [1], achieving ambitious GVAP targets will require additional financial commitment. One of the primary challenges in garnering these commitments lies in the difficulty of estimating the resources required for NIPs. Estimating the level of financing necessary to continue to reduce vaccine-preventable disease burden worldwide is important to sustain and improve upon advances made in immunization. Economic evidence is therefore essential to enable governments and development partners to project funding needs and commit resources toward vaccination.

This analysis augments the Decade of Vaccine Economics (DOVE) project [2], [3], [4], extending prior efforts to examine financing for immunization. Specifically, we explore six scenarios on the impact of future cost and financing projections, link the latest cost and financing requirements at a global scale, examine financing requirements by cost components (vaccine, supply chain, service delivery), and provide uncertainty ranges through sensitivity analyses. By linking detailed vaccine program costs [2] with a range of financing projections, this work provides an in-depth view of immunization program resource requirements that reflect dynamic realities such as economic growth and pricing policy changes over time.

## 2 Materials and methods

### 2.1 Analysis scope and approach

This analysis estimates the projected available financing and resulting funding gap based on immunization program costs across 94 low- and middle-income countries (LMICs) over 5 years, 2016–2020. This includes 73 countries supported by Gavi, the Vaccine Alliance, and 21 countries not eligible for Gavi support. Gavi countries include 36 low-income countries, 17 countries above the World Bank’s low-income threshold that received Gavi support, and 20 countries that will graduate from receiving Gavi support during 2016–2020 (see Appendix A for the full list of countries).

The model incorporates the following 18 vaccines currently in national immunization programs in some LMICs: Bacillus Calmette-Guérin (BCG), cholera, diphtheria-tetanus-pertussis (DTP), DTP-hepatititis B-hib (pentavalent),^2^ hepatitis B birth dose, human papillomavirus (HPV), inactivated polio (IPV), Japanese encephalitis (JE), malaria, measles, measles-rubella (MR), measles-mumps-rubella (MMR), meningococcal conjugate A (MenA), oral polio (OPV), pneumococccal conjugate (PCV), rotavirus, typhoid, and yellow fever (YF). These include 12 vaccines where Gavi supports partial or full financing to facilitate new vaccine introductions, five other traditional vaccines, and a cholera stockpile. These vaccines are delivered through a combination of channels, where 17 vaccines are provided through routine immunization programs and 8 vaccines through supplementary immunization activities (SIAs).^3^ The cholera vaccine is not routinely delivered but rather set aside as stockpiles for cholera control and outbreak response.

We estimate vaccine program costs for three mutually-exclusive components of routine immunization (vaccines, supply chain, and service delivery) and two components of SIAs (vaccines and operational support). *Vaccine costs* were defined as the costs of procuring vaccines and related injection equipment and safety boxes needed to safely administer vaccines. *Supply chain costs* included the costs for immunization-specific and shared^4^ transportation, storage, and cold-chain specific labor. *Service delivery costs* encompassed the costs of immunization-specific and shared^5^ personnel and non-personnel components including program management, training, social mobilization, surveillance, and other recurrent costs related to vaccination. *Operational support* for SIAs included vaccine delivery and management of vaccination campaign efforts. The categorization reflects classifications and definitions used in previous vaccine program analyses [5], [6], [7]. Projected available financing was estimated for each of these components.

Our method of analysis was developed with consideration of the approaches and lessons learned from previous immunization program costing exercises [5], [6], [7], [8], and the availability and use of country-level data. For example, data from national comprehensive multi-year plans (cMYPs) provided the basis for estimates of immunization costs and financing for the majority of Gavi countries. The cost and financing estimates in the cMYP are provided by country teams using standardized guidelines developed by the World Health Organization (WHO) [9]. Transparency of the model design was also valued for continuous use and ability to update future inputs. Table 1 presents the full scope of costing and financing analysis. All cost and financing projections are presented in constant 2010 US dollars (US$2010).

### 2.2 Costing of national immunization programs

The costing, financing, and funding gap analyses were conducted concurrently across 18 vaccines over the decade, 2011–2020. The costing analysis is presented in a companion piece [2]. Specifically, vaccine costs were primarily derived from Gavi, the Pan American Health Organization (PAHO), and UNICEF price projections [10], [11], [12], [13], [14], [15] in combination with doses from Gavi’s Adjusted Demand Forecast version 9 (ADFv9, released spring 2014) [16]. Supply chain costs were modeled based on simulations of country-specific models developed by the HERMES (Highly Extensible Resource for Modeling Event-Driven Supply Chains) Logistics Modeling Team [17], [18], [19], [20], [21], [22], [23], [24], [25], [26], [27]. Average and marginal service delivery costs per dose and operational support for SIAs were obtained from an analysis of cMYPs [28]. See Portnoy et al. for further details on costing methods and results [2].

### 2.3 Financing of national immunization programs

The financing analysis focuses on three funding sources: funds from country governments, funds from Gavi and funds from “other development partners” (ODP). Sources from ODP include multilateral and bilateral donors, non-governmental organizations, foundations, and private philanthropy organizations. Financing approaches are summarized in Table 2.

#### 2.3.1 Government financing

In Gavi-supported countries, we estimated governments’ vaccine co-financing obligations articulated in the Gavi co-financing policy based on the 2014 demand forecast (ADFv9) [16], [29]. We assumed that governments will meet these co-financing obligations for Gavi-supported vaccines [29]. For non-Gavi supported vaccines, the baseline percentage of government financing for vaccines was obtained from cMYPs and applied to the total estimated annual costs for those vaccines to estimate a plausible proportion of vaccine financing by government. If a cMYP was not available for a country, the percentage of government financing of routine vaccines was taken from the WHO/UNICEF Joint Reporting Form (JRF), and was projected forward until 2020 using a five-year rolling average [30]. Appendix A notes data sources for all countries in the analysis.

In response to the anticipated introduction of the inactivated polio vaccine (IPV) in these 94 countries as part of the polio eradication initiative [31], [32], governments in all 94 countries were assumed to cover a share of IPV routine vaccine financing according to the ratio of the price of the oral polio vaccine (OPV) relative to the price of IPV.

Proportions of government financing for supply chain and service delivery were obtained from the most recently validated year in a cMYP (called the “baseline year” in the cMYP) and applied to the same year’s estimates of supply chain and service delivery costs. In the 31 countries without cMYP data, population-weighted government financing ratios for supply chain and service delivery were estimated from a cMYP analysis of data for 63 countries. Government financing of supply chain and service delivery was projected to grow from the baseline year at the same rate as real gross domestic product (GDP) growth in the base case estimate. The cost of shared personnel was assumed to be 100% government-financed in the analysis; thus by default there is no funding gap estimated for shared personnel as we expect governments to fully fund human resources not specific to immunization.

For SIA vaccines without Gavi support, specifically the measles-mumps-rubella (MMR) vaccine and OPV, percentage of government financing relative to resource requirements was abstracted from cMYPs for the most recently validated year. This percentage was used to estimate government SIA vaccine and operational support financing across the decade. Specifically, countries with a funding gap in their baseline year of cMYP maintained the percentage gap in projected years. In the 31 countries without a cMYP, SIA government financing per capita metrics were obtained from 63 countries with cMYP data to project SIA government support. A full list of countries and their cMYP availability can be found in Appendix A. Metrics were population-weighted and vaccine-specific. For the 31 countries, no funding gap was projected for the SIA vaccine component due to a lack of data on any existing or future funding shortages.

#### 2.3.2 Gavi financing

We modeled financing amounts committed by Gavi based on routine and supplementary vaccine co-financing obligations according to Gavi’s demand forecast (ADFv9) between 2016 and 2020 [16]. These obligations were assumed to be met in full by Gavi over the decade. Gavi entirely finances all SIA vaccine doses for Gavi-supported vaccines in the 73 Gavi countries.

Gavi’s health systems strengthening (HSS) disbursements for 2011–2014 were used to project 2016–2020 disbursements. These disbursements were projected according to the ceiling set by Gavi for HSS support in each country relying on an assumption that the annual proportion of the support ceiling would be uniform until 2020 [33], [34], [35]. In addition, Gavi support for vaccine introduction was calculated by multiplying the vaccine introduction subsidy – US$0.80 for child vaccines and $2.40 for human papillomavirus (HPV) vaccine – per target person in the year of introduction for each vaccine [36]. Total Gavi non-vaccine support was separated into supply chain and service delivery components by disaggregating HSS proposal support according to the category of the approved Gavi grant. This analysis conducted by the Gavi Secretariat estimated 21% of HSS spending was going to supply chain and 79% to service delivery for all Gavi-approved HSS grants. Gavi’s operational support for SIAs was estimated by multiplying Gavi’s operational subsidy of $0.65 per target person by the target population of each Gavi-supported SIA campaign [36].

#### 2.3.3 Other Development Partner (ODP) financing

For 63 countries with cMYP data, we obtained the baseline year share of ODP financing relative to total resource requirements for each cost component – routine vaccine, routine supply chain, routine service delivery, SIA vaccine, and SIA operational support. For 31 countries without cMYP data, the percentage of ODP financing for each cost component was estimated based on an analysis of cMYP baseline year data abstracting population-weighted ODP financing ratios for supply chain and service delivery.

For routine vaccine, SIA vaccine, and SIA operational support components, the share of ODP financing was held constant across the decade in the base case. For routine supply chain and routine service delivery financing, ODP financing from the baseline year was projected to grow at the rate of real GDP growth for each country to account for inflation.

### 2.4 Funding gap of national immunization programs

The funding gap was obtained by taking the difference between estimated costs of providing vaccines and the financing projected to be available for the period from 2016 to 2020. This analysis presents the funding gap by financing source (country governments, Gavi, and ODP) as well as by cost component categories (vaccine, supply chain, and service delivery).

### 2.5 Scenario analyses

A series of scenario analyses were carried out to examine the impact of various cost and financing projections on an estimated funding gap. Specifically, we modeled six funding gap scenarios involving three costing scenarios and three financing scenarios.

Three costing scenarios were developed to modify base case cost estimates using the following methods:

1. Vaccine price reduction scenario: In this scenario, all 21 non-Gavi countries were hypothetically assumed to be able to access vaccines at Gavi-subsidized prices for all Gavi vaccines [10]. This demonstrates the impact on costs if additional middle-income countries were able to access the same vaccine prices as Gavi countries.
2. 90% coverage scenario: This scenario modeled the cost implications of reaching the GVAP goal for all countries to achieve 90% coverage by 2020 for vaccines included in their NIPs. In order to model this coverage increase, 2012 coverage levels were used to project coverage linearly for the 2016–2020 time period to eventually reach 90% coverage by 2020 for all vaccines that had been introduced prior to 2017. When the Gavi Strategic Demand Forecast version 9 (SDFv9) coverage for any country in any year was greater than modeled coverage for the 90% scenario, the SDFv9 coverage level was used. SDFv9 coverage was maintained for all routine vaccines introduced during or after 2017 [37], [38].
3. Marginal service delivery cost scale-up scenario: This scenario examined the additional costs of reaching hard to reach populations. Specifically, we applied a proportional increase (approximately 126%) to marginal service delivery costs for countries with DTP3^6^ coverage at or above 90% based on an estimate of cost per health center visit [39].

Three additional financing scenarios were modeled which altered base case financing projections using various methods:

4. Historic GDP elasticity of government financing: This scenario considered an optimistic government financing growth using estimates of the change in government health expenditures (GHE) relative to changes in GDP to predict vaccine financing trends in countries as their economies grow. All GHE and GDP data were obtained from the World Bank [40]. Elasticity was defined as the annual proportional change in GHE, divided by the same annual proportional change in GDP, estimated from 2009 to 2013. Results were aggregated by World Bank income group.
5. Projected government expenditures as a percentage of GDP financing: This scenario grew government financing based on government expenditure (GE) projections rather than real GDP growth [40].
6. Historic GDP elasticity of ODA financing: In this analysis, we accounted for change in ODP financing by examining changes to financing of other development assistance (ODA) grouped by WHO region [40]. The analysis estimated historic ODA elasticity of GDP to predict how ODP may decrease financing as recipient countries’ economies grow. Elasticity was defined as the annual proportional change in ODA, divided by the same annual proportional change in GDP, 2009–2013. Results were aggregated by WHO country regions.

### 2.6 Sensitivity analysis

Sensitivity analysis was carried out varying seven key costing inputs and one financing input in model simulations to construct the 95% uncertainty ranges around baseline estimates. Specifically, we performed a probabilistic sensitivity analysis using the Monte Carlo method, building distributions around key model parameters including total doses, vaccine prices, slopes and extrapolations of supply chain costs from reference countries, average and marginal cost per dose for service delivery, and operational cost per dose for SIA for costing and real GDP growth for financing. For each parameter, random draws were taken from specified distributions 10,000 times. Non-cost values were given a beta distribution, while cost values used gamma distributions in order to represent the skew of observed costing data. Costing parameters were ranged between half and double the base estimate to examine the effect of each variable; real GDP growth was varied from 91% to 109% capturing two standard deviations across all countries and years of data. This analysis was implemented using the latest version of the Palisade’s @RISK software.

## 3 Results

### 3.1 Base case

In the remaining five years of this decade (2016–2020), we observe a base case funding gap of $7.6 billion (95% uncertainty range: $4.6–$11.8 billion), resulting from the difference between an estimated US$35.7 billion in NIP costs and US$28.1 billion in projected available financing. This funding gap consisted of $6.9 billion across 73 Gavi countries and $0.7 billion among the 21 non-Gavi countries. Service delivery to support immunization programs, including costs for program management, training, social mobilization, and surveillance accounted for the largest proportion of the expected costs ($14.0 billion) and contributed 65% or US$5.0 billion ($2.7–$8.4 billion) to the funding gap. A funding gap of US$1.1 billion for vaccines ($0.9–$2.7 billion) and $1.5 billion for supply chain ($1.1–$2.0 billion) comprised 9% and 66% of estimated costs respectively for those components. Table 3 shows the cost, financing, and funding gap over 2016–2020 by component. While service delivery is the main driver of the funding gap in absolute dollar value, the supply chain component faces the greatest gap in funding as a proportion of costs.

**Fig. 1:**
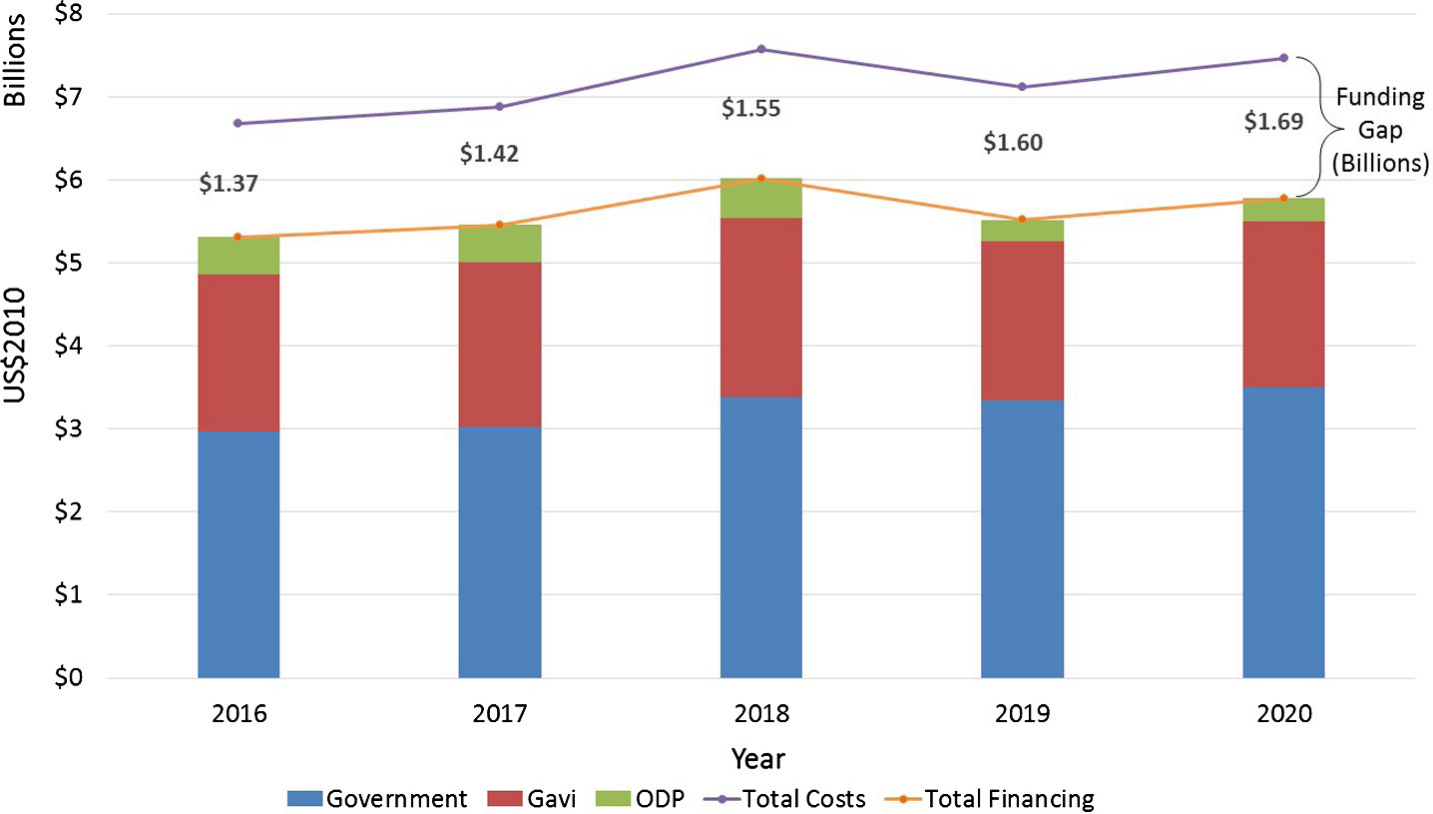
Projected available costs, financing and funding gap by funding source by year.

The base case financing highlights the important role of government financing with $16.2 billion contributed by national governments. External donors continue to play a supportive role, with $9.9 billion provided by Gavi and $1.9 billion by ODP between the years 2016–2020. Fig. 1 presents the total projected available financing for immunization programs by funding source across the years.

### 3.2 Scenario and sensitivity analyses

Among the modeled scenarios, the funding gap was found to be smallest for the vaccine price reduction scenario ($6.3 billion) and highest for the 90% coverage scenario ($9.2 billion). Scaling up marginal delivery costs to reach hard-to-reach populations had the next largest effect, increasing the funding gap by 13% ($1.0 billion). If non-Gavi countries were able to access Gavi vaccine prices the funding gap was reduced by 19% ($1.4 billion) compared to the base case. The costing scenarios had a greater effect on the funding gap ranging the final funding gap estimate by 81–119% ($6.25–$9.16 billion) compared to financing scenarios ranging the funding gap by 96–101% ($7.39–$7.68 billion). Table 4 presents the results across examined scenarios.

The three financing scenarios had little effect on the base funding gap estimate. Specifically, changing the projections from real GDP growth to government health expenditure elasticity of GDP closed the funding gap by nearly four percent to $7.4 billion over 2016–2020. Growing government financing based on government expenditure projections or altering ODP growth using GDP elasticity of ODA financing had a minimal effect on the funding gap.

Sensitivity analysis around base case parameter values revealed that the service delivery marginal cost per dose is the biggest driver of the base funding gap estimate (24% lower to 41% higher), followed by projected doses (29% lower to 33% higher), service delivery average cost per dose (7% lower to 15% higher), and vaccine prices (8% lower to 13% higher). Assumptions around real GDP growth for financing projections, SIA operational cost per dose, and supply chain cost extrapolations were less influential on the funding gap (see Appendix B for a tornado diagram).

## 4 Discussion

Despite the commitments from government, Gavi, and other development partners to achieve global immunization goals, we estimate a $7.6 billion (uncertainty range: $4.6–$11.8 billion) base case funding gap from 2016 to 2020 for NIPs. This funding gap amounts to approximately 0.2% of general government expenditures on average across 94 countries. Taking government health expenditures as a percentage of government expenditures from 2012–2014 and applying it to 2016–2020, the funding gap represents 2.3% of government health expenditures on average across 94 countries. This analysis conducted a series of scenario analyses to examine the impact of projections on financing and funding gap for NIPs to reach GVAP targets in the Decade of Vaccines. We found that the financing scenarios, which optimistically grew available government financing or pessimistically grew ODP financing, did not have a large impact on closing the funding gap. This suggests that precision of costing data may be more essential than the financing data to estimate the funding gap. It also speaks to the need for changes in immunization program investment beyond the level that was projected at the beginning of the decade to address this funding gap for immunizations.

Of note, the main driver of the funding gap stems from the service delivery component, which highlights the critical need for health systems strengthening efforts to support immunization coverage goals. Although the overall projected cost was smallest for supply chains ($2.3 billion from 2016 to 2020), this component was underfunded, covering 34% of total costs. Note that this analysis was done prior to the launch of Gavi’s Cold Chain Equipment (CCE) facility which will provide up to $50 million in support. As for sources of financing, the majority of available financing (58%) was projected to come from country governments. This finding is similar to that found in a recent multi-country study of routine immunization financing (EPIC) and analysis of cMYP data [1], [41], [42]. While this supports the GVAP principles to promote country ownership in national immunization programs, additional government funds may be necessary both to reduce the funding gap and reliance on support from Gavi and other development partners [43].

In 2015, Gavi replenished its commitment to deliver vaccines to millions of the world’s poorest children with a $7.5 billion pledge for 2016–2020 from its partners, including international institutions, donor organizations, country governments, and the pharmaceutical industry. These new pledges support Gavi’s financing projected in this analysis and are a part of the $9.9 billion that Gavi is projected to contribute towards immunization over the next five years. This contribution from Gavi, providing 35.4% of projected total funding, is an essential piece of the financial support structure for low- and lower-middle income countries that will enable countries to immunize an additional 300 million children and avert 5–6 million premature deaths [44]. Since this analysis, Gavi and the Global Polio Eradication Initiative (GPEI) have also committed to supporting a one-time cash grant to cover additional costs of introducing the IPV vaccine for Gavi countries. If polio is eradicated between 2016 and 2020, there may also be opportunities to reallocate some funding to meet the funding gap.

Our analysis updates an earlier estimate conducted by Gandhi et al. (2013). Our analysis projected financing from governments, Gavi, and other development partners to be $28.1 billion, greater than in the previous estimate by approximately $9.4 billion between 2016 and 2020 [6]. This is due to a distinct methodological approach based on proportional rather than per capita allocations. As a result, our analysis estimates a smaller funding gap of $7.6 billion compared to $14.2 in the previous analysis. This could be due to use of more up-to-date data supporting actual increases in financing in the decade resulting in a smaller gap. While the projection methods for financing differ between the two analyses, the growth in the financing estimates is also due to a combination of greater immunization coverage scale-up and increasing number of vaccine introductions across the decade in the latest Gavi demand forecast [16] compared to the version used by Gandhi et al. in their analysis [45]. Financing projections will grow over time as hard-to-reach populations may become more costly to access and new vaccine introductions demand greater commitment. A comparison to the previous analysis by Gandhi et al. (2013) can be found in Table 5.

There are a number of limitations in this analysis that are important to note. Specifically, the costs, financing, and funding gap estimates presented are subject to uncertainties regarding changes to future vaccine prices, doses demanded and procured, and financial flows. While we tried to use a series of scenario and sensitivity analyses to understand the impact of current data and assumptions, additional scenarios, budgetary data, and empirical evidence could be examined to look further into these projections. For example, the uncertainty around Gavi’s demand forecast is important to note as vaccine recommendations and policies around new vaccines are in a state of rapid change. In addition, model inputs such as vaccine prices, demand forecasts, government expenditure data, population data and co-financing groupings are constantly refined by Gavi and others. The uncertainty ranges could also be improved with additional data to reflect different uncertainties of inputs. While our results used the latest data sources at the time of analysis, we have built the model such that these estimates could be updated in future analyses.

The quality and availability of existing data is another limiting factor in this analysis, particularly our reliance on cMYP data. While cMYP data currently are the best sources for costing and financing of immunizations across LMICs, they are compiled for Gavi applications with potential inconsistencies in cost definitions and calculations, and weak validation methods. In addition, cMYP data were only available in two-thirds of countries receiving Gavi support for immunization, which were then used to estimate costs and financing for the remaining countries. Since some countries without cMYPs are higher-income countries, the base case may be an underestimation of costs and government financing and an overestimation of contributions of other development partners, adding to uncertainties around the results. However, comparisons with latest evidence on vaccine assistance across all LMICs suggest that our projection of contributions by other development partners may be conservative [43]. Even with the variations in data quality, cMYPs are currently the most detailed source of continuously updated cost and financing estimates of NIPs at a country level.

This analysis informs the greater financial support necessary to support vaccine programs across 94 countries to achieve the GVAP vision to make the benefits of immunization available to all. The estimated funding gap, regardless of scenarios, suggests the need for additional country and donor commitments to mobilize and effectively allocate resources, especially for the service delivery component of national immunization programs and relatively underfunded country supply chains. Further research on the influences that investments in technological changes and price reductions have on reducing the funding gap should be conducted to inform governments and other development partners about the most efficient use of future resources.

## Figures and Tables

**Table 1:** Scope of analysis by delivery mechanism, components, vaccines, and sources.^a^

Components	Vaccines with Gavi support (Financed by Gavi, government & other development partners)	Vaccines without Gavi support (Financed by government & other development partners)
*Routine*		
Vaccine • Vaccine incl. freight • Injection equipment and safety boxes	DTP-HepB-Hib, HPV, IPV, JE, Malaria, Measles 2nd, MR, MenA, PCV, Rotavirus, Typhoid, YF	BCG, DTP, HepB birth, Measles 1st, MMR, OPV
Supply chain • Immunization-specific transportation • Shared transportation • Storage • Labor		
Service delivery • Immunization-specific personnel • Shared personnel • Non-personnel incl. training, surveillance, program management, social mobilization		

*SIA*		
Vaccine • Vaccine incl. freight • Injection equipment and safety boxes	JE, Malaria, Measles, MR, MenA, Typhoid, YF	MMR, OPV
Operational support • Personnel • Other operational costs incl. training, transportation, and social mobilization		

a The analysis also includes a cholera stockpile, additional support for Gavi supported vaccine stockpiles, and advanced market commitments (AMC) for PCV.

**Table 2:** Summary of financing estimation and projection methods.

Government, Gavi, or ODP	Routine or SIA	Vaccines	Country groupings^a^	Component	Base financing	Projection method	Implications on the funding gap
Government financing	Routine	Gavi supported routine vaccines	Gavi countries (73)	Vaccine	Gavi co-financing obligations specified by Gavi policy and ADFv9^b^	Dependent on co-financing obligations specified in ADFv9	All government commitments forecasted by the ADFv9 were projected to be met in full
		Routine vaccines not supported by Gavi	Gavi countries with cMYP data (63)		Percentage of government financing from cMYP baseline year data	Constant percentage applied to estimated costs for projection years	
		Gavi supported routine vaccines	Non-Gavi countries (21)		Percentage of government financing from JRF data	Five-year rolling average	Baseline year levels of government financing will remain constant over time relative to projected costs
		Routine vaccines not supported by Gavi	Countries without cMYP data (31)				
		All routine vaccines	Gavi countries with cMYP data (63)	Supply chain and service delivery^c^	Percentage of government financing from cMYP baseline year data	IMF projections of real GDP growth	Government immunization budgets will grow at the same rate as GHE, which is assumed to grow at real GDP rates
			Countries without cMYP data (31)		Population-weighted government financing ratios estimated from cMYP baseline year data		
	SIA	All SIA vaccines	Gavi countries with cMYP data (63)	Vaccine & Operational	Percentage of government financing from cMYP baseline year data	Constant percentage applied to estimated costs for projection years	Only countries with a funding gap in their baseline year cMYP data have an estimated funding gap for the SIA vaccine & operational components
			Countries without cMYP data (31)	Vaccine & Operational	Population-weighted government financing ratios estimated from cMYP baseline year data		There is no estimated funding gap for the SIA vaccine & operational components

Gavi financing	Routine	Gavi supported routine vaccines	Gavi countries (73)	Vaccine	Gavi co-financing obligations specified by Gavi policy and ADFv9	Dependent on co-financing obligations specified in ADFv9	All Gavi commitments forecasted by the ADFv9 were projected to be met in full
				Vaccine introduction support	Gavi vaccine introduction support subsidy ($0.80 for infant vaccines and $2.40 for HPV)	Calculated by multiplying the vaccine introduction subsidy per target person in year of introduction for each Gavi-supported vaccine	Gavi will provide funding in line with its vaccine introduction grant policy
				Supply chain and service delivery	Gavi health systems strengthening (HSS) disbursements	Projected according to ceilings set by Gavi for HSS support	Gavi HSS support will not exceed ceiling levels across the decade
	SIA	Gavi supported SIA vaccines		Vaccine	Gavi co-financing obligations specified by Gavi policy and ADFv9	Dependent on co-financing obligations specified in ADFv9	Gavi entirely finances all SIA vaccines doses for Gavi-supported vaccines in Gavi countries
				Operational	Gavi operational support subsidy ($0.65 per target person)	Calculated by multiplying the operational support subsidy per target person by target population of each Gavi-supported campaign	Gavi will provide funding in line with its operational support for campaigns policy

ODP financing	Routine	All routine vaccines	Gavi countries with cMYP data (63)	Vaccine	Percentage of ODP financing from cMYP baseline year data	Percentage applied to estimated costs for projection years	Baseline year levels of ODP financing will remain constant over time relative to projected costs
				Supply chain and service delivery		IMF projections of real GDP growth	ODP immunization support will grow at least at the same rate as economic growth
			Countries without cMYP data (31)	Vaccine	Population-weighted ODP financing ratios estimated from cMYP baseline year data	Percentage applied to estimated costs for projection years	There is no estimated funding gap for the routine vaccine component for countries without cMYP data
				Supply chain and service delivery		IMF projections of real GDP growth	
	SIA	All SIA vaccines	Gavi countries with cMYP data (63)	Vaccine & Operational	Percentage of ODP financing from cMYP baseline year data	Percentage applied to estimated costs for projection years	Only countries with a funding gap in their baseline year cMYP data have an estimated funding gap for the SIA vaccine & operational components
			Countries without cMYP data (31)	Vaccine & Operational	Population-weighted ODP financing ratios estimated from cMYP baseline year data		There is no estimated funding gap for the SIA vaccine & operational components

Abbreviations: cMYP, comprehensive multi-year plan; GDP, gross domestic product; HPV, human papillomavirus vaccine; HSS, health systems strengthening; IMF, International Monetary Fund; JRF, WHO-UNICEF Joint Reporting Form; MMR, measles-mumps-rubella vaccine; ODP, other development partners; OPV, oral polio vaccine; SIA, supplementary immunization activities.

a See Appendix A for country groupings. Projected Gavi support for vaccines was dependent on country-specific forecasts of vaccine introduction and annual anticipated Gavi status. Gavi vaccines include the following: DTP-HepB-Hib (Pentavalent), HPV, IPV, JE, malaria, measles 2nd dose and measles SIAs, MR, MenA, PCV, rotavirus, typhoid, YF, and cholera (stockpile). Vaccines without Gavi support include BCG, DTP, HepB birth dose, measles 1st dose, MMR, and OPV.

b As introduction of inactivated polio vaccine (IPV) is anticipated in all 94 countries as part of the polio eradication plan, a separate assumption was developed for the financing of the routine IPV rollout. Governments in all 94 countries are assumed to cover a share of IPV routine vaccine financing according to the ratio of the OPV vaccine price relative to the IPV vaccine price. The remaining price differential is included in the funding gap estimate.

c The cost of shared personnel is assumed to be 100% financed by all financing sources (i.e., by default there is no funding gap for shared personnel). Therefore, all shared personnel costs that are not met by Gavi and ODP financing are projected to be funded fully by government financing.

**Table 3:** Estimated costs, projected financing, and resulting funding gap for 2016–2020 (US$2010 billions).

Component	Costs	Financing	Funding gap
**Routine**	**$30.9**	**$23.4**	**$7.5**
Vaccine	$12.3	$11.2	$1.1
Supply chain	$2.3	$0.8	$1.5
Service delivery	$16.3	$11.4	$4.9
**SIA**	**$4.8**	**$4.6**	**$0.1**
Vaccine	$2.1	$2.0	<$0.1
Operational support	$2.6	$2.6	<$0.1
**Total**	**$35.7**	**$28.1**	**$7.6**

**Table 4:** Funding gap scenario results, 2016–2020 (US$ billions).

Scenario #	Scenarios	Total costs	Total financing	Total funding gap	% Change in funding gap from base case
	**Base case**	35.69	28.06	7.63	N/A
	**Costing Scenarios (using base financing)**				
1	Price Reduction Scenario Costs	34.26	28.06	6.20	−18.76%
2	90% Coverage Scenario Costs	37.46	28.06	9.41	23.22%
3	Marginal Service Delivery Cost Scale-Up	36.71	28.06	8.65	13.32%

	**Financing Scenarios (using base costs)**				
4	Historic GDP Elasticity of Government Financing	35.69	28.30	7.39	−3.79%
5	Projected Government Expenditures as % of GDP Financing	35.69	28.01	7.68	0.01%
6	Historic GDP Elasticity of ODA Financing	35.69	28.02	7.67	−0.01%

**Table 5:** Comparison to Gandhi et al. [6] Findings, 2016–2020 (US$2010 billions).

	Gandhi et al.^a^	Our analysis
Total costs	32.9	35.7
Total financing	18.7	28.1
Total funding gap	14.2	7.6

a Key methodological differences with the Gandhi et al. analysis include the following. Gandhi et al. uses: (1) dose projections from 2011 Gavi Adjusted Demand Forecast (version 4) rather than from 2014 (version 9); (2) constant vaccine prices across the decades rather than prices that are projected to decrease; (3) a global deflator for conversion to US$2010 rather than local inflation based on consumer price indices (CPI); (4) different cMYP data and methods used to derive projected available financing (see Table 1 for current analysis and Table 3 in Gandhi et al.); (5) earlier values of Gavi support subsidies; and (6) financing metrics based on Gavi co-financing groupings (see Table 3 in Gandhi et al.).

## Footnotes

2 DTP-HepB-Hib refers to the diphtheria-tetanus-pertussis-hepatitis B-Haemophilus influenzae type b vaccine, also known as pentavalent.

3 *Supplementary immunization activities (SIAs)* are defined through accelerated disease control (ADC), elimination, or eradication initiatives (e.g., polio eradication campaigns) that go beyond routine immunization.

4 Shared transportation refers to any transportation which is utilized for multiple purposes, including but not limited to immunization.

5 Shared personnel are defined as resources that are not specific to immunization and are used by different programs or activities in the health sector.

6 DTP3 includes three doses of diphtheria, tetanus and pertussis vaccines and is often used as a standard measure for the strength of a country’s immunization system.
