# Supplementary material for: Funding gap for immunization across 94 low- and middle-income countries
Source: Vaccine. 2016 Dec 7;34(50):6408–16. doi: 10.1016/j.vaccine.2016.09.036 (PMC5142419; doi:10.1016/j.vaccine.2016.09.036)
Supplement: Supplementary data 1 [file mmc1.docx]

**Funding gap for immunization across 94 low- and middle-income countries**

**Appendix A**: Country Reference and cMYP Baseline Year for Baseline Financing

| **Country** | **WHO Region** | **World Bank Income Group** | **Gavi**  **Co-Financing Status** | **cMYP Baseline Year** | **Additional cMYP Considerations** |
| --- | --- | --- | --- | --- | --- |
|  |  | **2015** | **2014** |  |  |
| Afghanistan | EMRO | LIC | Low-Income | 2009 |  |
| Angola | AFRO | UMIC | Graduating | 2010 |  |
| Armenia | EURO | LMIC | Graduating | 2009 |  |
| Azerbaijan | EURO | UMIC | Graduating | 2009 |  |
| Bangladesh | SEARO | LMIC | Low-Income | 2004 | *non-recent cMYP used following data quality checks* |
| Belize | AMRO | UMIC | Not Eligible | Not Available | *no cMYP available* |
| Benin | AFRO | LIC | Low-Income | 2008 |  |
| Bhutan | SEARO | LMIC | Graduating | 2006 |  |
| Bolivia | AMRO | LMIC | Graduating | Not Available | *cMYP not used following data quality checks* |
| Burkina Faso | AFRO | LIC | Low-Income | 2010 |  |
| Burundi | AFRO | LIC | Low-Income | 2010 |  |
| Cambodia | WPRO | LIC | Low-Income | 2007 |  |
| Cameroon | AFRO | LMIC | Intermediate | 2010 |  |
| Cape Verde | AFRO | LMIC | Not Eligible | Not Available | *cMYP not used following data quality checks* |
| Central African Republic | AFRO | LIC | Low-Income | 2010 |  |
| Chad | AFRO | LIC | Low-Income | 2011 |  |
| Comoros | AFRO | LIC | Low-Income | 2005 |  |
| Congo, Dem. Rep. | AFRO | LIC | Low-Income | 2010 |  |
| Congo | AFRO | LMIC | Graduating | 2010 |  |
| Cote d'Ivoire | AFRO | LMIC | Intermediate | 2009 |  |
| Cuba | AMRO | UMIC | Graduating | Not Available | *cMYP not used following data quality checks* |
| Djibouti | EMRO | LMIC | Intermediate | 2010 |  |
| Egypt | EMRO | LMIC | Not Eligible | Not Available | *no cMYP available* |
| El Salvador | AMRO | LMIC | Not Eligible | Not Available | *no cMYP available* |
| Eritrea | AFRO | LIC | Low-Income | 2011 |  |
| Ethiopia | AFRO | LIC | Low-Income | 2011 |  |
| Fiji | WPRO | UMIC | Not Eligible | Not Available | *no cMYP available* |
| Gambia | AFRO | LIC | Low-Income | 2010 |  |
| Georgia | EURO | LMIC | Graduating | 2010 |  |
| Ghana | AFRO | LMIC | Intermediate | Not Available | *cMYP not used following data quality checks* |
| Guatemala | AMRO | LMIC | Not Eligible | Not Available | *no cMYP available* |
| Guinea | AFRO | LIC | Low-Income | 2010 |  |
| Guinea-Bissau | AFRO | LIC | Low-Income | 2009 |  |
| Guyana | AMRO | LMIC | Graduating | Not Available | *cMYP not used following data quality checks* |
| Haiti | AMRO | LIC | Low-Income | 2006 |  |
| Honduras | AMRO | LMIC | Graduating | Not Available | *cMYP not used following data quality checks* |
| India | SEARO | LMIC | Intermediate | 2011 |  |
| Indonesia | SEARO | LMIC | Graduating | 2009 |  |
| Iraq | EMRO | UMIC | Not Eligible | Not Available | *no cMYP available* |
| Kenya | AFRO | LMIC | Low-Income | 2010 |  |
| Kiribati | WPRO | LMIC | Graduating | 2010 |  |
| Korea, DPR | SEARO | LIC | Low-Income | 2010 |  |
| Kosovo | EURO | LMIC | Not Eligible | Not Available | *no cMYP available* |
| Kyrgyzstan | EURO | LMIC | Low-Income | 2009 |  |
| Lao PDR | WPRO | LMIC | Intermediate | 2010 |  |
| Lesotho | AFRO | LMIC | Intermediate | 2010 | *non-recent cMYP used following data quality checks* |
| Liberia | AFRO | LIC | Low-Income | 2009 |  |
| Madagascar | AFRO | LIC | Low-Income | 2010 |  |
| Malawi | AFRO | LIC | Low-Income | 2008 |  |
| Mali | AFRO | LIC | Low-Income | 2009 |  |
| Marshall Islands | WPRO | UMIC | Not Eligible | Not Available | *no cMYP available* |
| Mauritania | AFRO | LMIC | Intermediate | 2010 |  |
| Micronesia | WPRO | LMIC | Not Eligible | Not Available | *no cMYP available* |
| Moldova | EURO | LMIC | Graduating | 2009 |  |
| Mongolia | WPRO | UMIC | Graduating | Not Available | *cMYP not used following data quality checks* |
| Morocco | EMRO | LMIC | Not Eligible | Not Available | *no cMYP available* |
| Mozambique | AFRO | LIC | Low-Income | 2011 |  |
| Myanmar | SEARO | LMIC | Low-Income | 2011 |  |
| Nepal | SEARO | LIC | Low-Income | 2010 |  |
| Nicaragua | AMRO | LMIC | Graduating | Not Available | *cMYP not used following data quality checks* |
| Niger | AFRO | LIC | Low-Income | 2009 |  |
| Nigeria | AFRO | LMIC | Intermediate | 2008 |  |
| Pakistan | EMRO | LMIC | Intermediate | 2008 |  |
| Papua New Guinea | WPRO | LMIC | Graduating | Not Available | *cMYP not used following data quality checks* |
| Paraguay | AMRO | UMIC | Not Eligible | Not Available | *no cMYP available* |
| Philippines | WPRO | LMIC | Not Eligible | Not Available | *no cMYP available* |
| Rwanda | AFRO | LIC | Low-Income | 2006 | *non-recent cMYP used following data quality checks* |
| Samoa | WPRO | LMIC | Not Eligible | Not Available | *no cMYP available* |
| Sao Tome and Principe | AFRO | LMIC | Intermediate | 2010 |  |
| Senegal | AFRO | LMIC | Intermediate | 2010 |  |
| Sierra Leone | AFRO | LIC | Low-Income | 2010 |  |
| Solomon Islands | WPRO | LMIC | Intermediate | Not Available | *cMYP not used following data quality checks* |
| Somalia | EMRO | LIC | Low-Income | 2010 |  |
| Sri Lanka | SEARO | LMIC | Graduating | 2006 | *non-recent cMYP used following data quality checks* |
| Sudan: North | EMRO | LMIC | Intermediate | 2010 |  |
| Sudan: South | AFRO | LMIC | Low-Income | 2011 |  |
| Swaziland | AFRO | LMIC | Not Eligible | Not Available | *cMYP not used following data quality checks* |
| Syria | EMRO | LMIC | Not Eligible | Not Available | *cMYP not used following data quality checks* |
| Tajikistan | EURO | LMIC | Low-Income | 2009 |  |
| Tanzania | AFRO | LIC | Low-Income | 2010 |  |
| Timor-Leste | SEARO | LMIC | Graduating | 2010 |  |
| Togo | AFRO | LIC | Low-Income | 2009 |  |
| Tonga | WPRO | UMIC | Not Eligible | Not Available | *no cMYP available* |
| Turkmenistan | EURO | UMIC | Not Eligible | Not Available | *no cMYP available* |
| Tuvalu | WPRO | UMIC | Not Eligible | Not Available | *no cMYP available* |
| Uganda | AFRO | LIC | Low-Income | 2011 |  |
| Ukraine | EURO | LMIC | Graduating | Not Available | *no cMYP available* |
| Uzbekistan | EURO | LMIC | Graduating | 2009 |  |
| Vanuatu | WPRO | LMIC | Not Eligible | Not Available | *no cMYP available* |
| Viet Nam | WPRO | LMIC | Intermediate | Not Available | *cMYP not used following data quality checks* |
| West Bank and Gaza | EMRO | LMIC | Not Eligible | Not Available | *no cMYP available* |
| Yemen | EMRO | LMIC | Intermediate | 2009 |  |
| Zambia | AFRO | LMIC | Intermediate | 2010 |  |
| Zimbabwe | AFRO | LIC | Low-Income | 2010 |  |

**Appendix B**: Elasticities estimated for financing scenarios

1. Historic elasticity of GDP by government health expenditure (GHE)

(Used to predict how countries may alter vaccine financing as their economies grow by income level)

| **Financing Scenario - GHE Elasticity*** | | | |
| --- | --- | --- | --- |
| **Income Level** | **n** | **Elasticity** | **p-value** |
| **LIC** | 32 | 1.9505778 | 0.5013 |
| **LMIC** | 43 | 1.60644 |  |
| **UMIC** | 9 | 0.9652083 |  |

*One way ANOVA on World Bank income level classification

1. Historic Official Development Assistance (ODA) elasticity of GDP

(Used to predict how other develop partner vaccine financing may alter as recipient countries’ economies grow)

| **Financing Scenario - ODA Elasticity*** | | | |
| --- | --- | --- | --- |
| **WHO Region** | **n** | **Elasticity** | **p-value** |
| **AFRO** | 37 | 1.2504728 | 0.4314 |
| **AMRO** | 9 | 0.0499724 |  |
| **EURO** | 9 | 0.0499724 |  |
| **EMRO** | 7 | 0.9778069 |  |
| **SEARO** | 8 | 0.9778069 |  |
| **WPRO** | 14 | 0.3149674 |  |

*One way ANOVA run as AFRO vs. AMRO+EURO vs. EMRO+SEARO vs. WPRO

**Appendix C**: Funding Gap Sensitivity analysis: Tornado diagram results*

**Total Doses**

**SD Marginal Cost per Dose**

**Financing – Real GDP Growth**

**SC Reference Country Slopes**

**SIA Operational Cost per Dose**

**SC Scalar Relationship**

**Vaccine Price**

**SD Average Cost per Dose**

**SD: Service delivery; SC: Supply chain.*
